# How biomarker patterns can be utilized to identify individuals with a high disease burden: a bioinformatics approach towards predictive, preventive, and personalized (3P) medicine

**DOI:** 10.1007/s13167-021-00255-0

**Published:** 2021-09-29

**Authors:** Nina Bertele, Alexander Karabatsiakis, Claudia Buss, Anat Talmon

**Affiliations:** 1 Psychology Department, Stanford University, Stanford, CA USA; 2 Institute of Medical Psychology, Charité – Universitätsmedizin Berlin, corporate member of Freie Universität Berlin and Humboldt-Universität zu Berlin, Berlin, Germany; 3 Institute of Psychology, Department of Clinical Psychology-II, University of Innsbruck, Innsbruck, Austria; 4 Development, Health and Disease Research Program, Department of Pediatrics, University of California Irvine, Irvine, CA USA

**Keywords:** Biomarker patterns, Personalized medicine (PPPM/3PM), Patient stratification, Risk assessment, Childhood maltreatment, Psychiatric disorders

## Abstract

Prevalences of non-communicable diseases such as depression and a range of somatic diseases are continuously increasing requiring simple and inexpensive ways to identify high-risk individuals to target with predictive and preventive approaches. Using *k*-mean cluster analytics, in study 1, we identified biochemical clusters (based on C-reactive protein, interleukin-6, fibrinogen, cortisol, and creatinine) and examined their link to diseases. Analyses were conducted in a US American sample (from the Midlife in the US study, *N* = 1234) and validated in a Japanese sample (from the Midlife in Japan study, *N* = 378). In study 2, we investigated the link of the biochemical clusters from study 1 to childhood maltreatment (CM). The three identified biochemical clusters included one cluster (with high inflammatory signaling and low cortisol and creatinine concentrations) indicating the highest disease burden. This high-risk cluster also reported the highest CM exposure. The current study demonstrates how biomarkers can be utilized to identify individuals with a high disease burden and thus, may help to target these high-risk individuals with tailored prevention/intervention, towards personalized medicine. Furthermore, our findings raise the question whether the found biochemical clusters have predictive character, as a tool to identify high-risk individuals enabling targeted prevention. The finding that CM was mostly prevalent in the high-risk cluster provides first hints that the clusters could indeed have predictive character and highlight CM as a central disease susceptibility factor and possibly as a leverage point for disease prevention/intervention.

**Supplementary Information:**

The online version contains supplementary material available at 10.1007/s13167-021-00255-0.

## Introduction

### The global burden of disease—current situation

Prevalence and incidence of non-communicable diseases (NCD) are continuously increasing in numbers, causing a strong socio-economic as well as a medical burden to the healthcare systems. Economically speaking, the US healthcare costs have steadily increased for 4 consecutive years, to reach 3.8 trillion US dollars in 2019 [1, 2]. NCD caused 90% of these costs as they result in massive long-term treatment costs and are often present with comorbidities [1, 2]. Thus, the prevention of NCD, and in this context the identification of at-risk individuals and sensitive biomarkers of disease risk, is more important than ever as it represents a leverage point to reduce the economic as well as the individual burden of diseases.

### The contribution of the current study

**Fig. 1:**
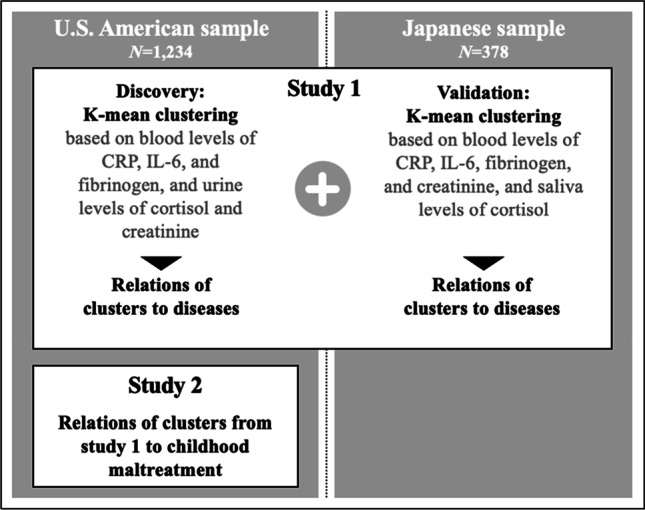
Study workflow chart. *Note*: CRP = C-reactive protein, IL-6 = Interleukin-6. All one-time measures except saliva cortisol in the Japanese sample which was averaged across three time points (morning, noon, evening) for a total of 3 days

The two-consecutive study presented here demonstrates how routinely assessed biomarkers can be bioinformatically clustered and utilized to identify individuals with a high disease burden. Specifically, in study 1, we employed a clustering approach based on C-reactive protein (CRP), interleukin-6 (IL-6), fibrinogen, cortisol, and creatinine concentrations in a US cohort and validated the identified clusters in a Japanese cohort (for a study overview, see Fig. 1). We then linked these biochemical clusters to documented diseases including depression, heart disease, hypertension, stroke, peptic ulcer disease (PUD), and cancer. In study 2, we tested the association of childhood maltreatment (CM), a well-established early-life risk factor for developing mental and somatic disorders, with diseases as well as with the identified biochemical clusters from study 1.

## General methods

### Description of the study populations

#### US American sample

Data were drawn from the biomarker subsample of the *Midlife in the United States* (MIDUS) study between 1995 and 1996 [3]. For more information about the project, please see http://www.midus.wisc.edu/data/index.php. A total of 1255 individuals participated in the biomarker study, and of those complete biomarker data was available from 1234 individuals.

#### Japanese Sample

Data were drawn from the *Midlife in Japan* (MIDJA) study (N = 1027). In 2009–2010 biomarker data were generated for a subset of these participants (*N* = 378). Data were obtained analogically to MIDUS.

## Study 1

### Introduction

#### The importance of risk evaluation in personalized medicine, targeted prevention, and predictive diagnostics

According to the Global Burden of Disease study (2017), between 1990 and 2017, disability-adjusted life years (DALYs) due to NCD increased from 1.2 to 1.6 billion. With that, NCD caused more than 60% of DALYs worldwide [4]. But NCD cause not only individual suffering but also burden society as a whole, due to massive monetary and non-monetary costs [4, 5]. Relying on interventions—no matter how effective they are—after individuals are already ill is therefore a pivotal fallacy. Instead, current developments require simple and inexpensive ways to identify high-risk individuals to target with both preventive and interventive approaches. Furthermore, it is increasingly becoming clear that many well-established risk factors (such as body mass index (BMI) outside the normal range [6], genetic risk factors [7, 8]) supposedly helping to identify individuals at high risk for certain diseases are not independently from the individual environment and do not behave the same way across different individuals, highlighting the importance of personalized, tailored approaches in the context of preventive medicine. The presence of one particular risk factor might not have much predictive character for negative outcomes without being considered systemically/holistically, that is, in the context of other physiological, environmental, psychological, and biochemical parameters and processes [e.g., 6–8]. Despite these intricacies, at the same time, disease-predictive measures should be cost-efficient making it possible to implement them in the healthcare system.

#### The allostatic load index: chances and limitations

One particular concept that has become well-established in the literature is the concept of allostatic load (referring to the cumulative burden of chronic stress and adverse life events) with its suggested allostatic load index (ALI) [9]. ALI is a cumulative multi-system risk score based on physiological and biochemical measures [10]. For each system, risk indices are calculated as the proportion of biomarkers for which an individual falls into predefined high-risk quartiles.

As a systemic risk score, ALI is predictive for various outcomes, including all-cause mortality [11, 12], while there are some critical limitations concerning its conceptualization. First, calculating a risk score as the sum of different system risk scores does not allow to account for intersystemic interactions and the possible predictive effect of these interactions. This gap is unfortunate as ALI includes parameters that indeed are not independent of each other, such as BMI and blood pressure [13]. Another concern refers to practicability and implementation of ALI into the healthcare system. While ALI considers parameters that can be assessed relatively simple, it is still likely that, for most individuals, parameters are only partially available, possibly limiting the predictive power of ALI. Together, ALI is a profound concept but artificially splits physiological processes that are woven into a holistic allostatic reaction, as acknowledged by the developers of ALI [14]. Furthermore, ALI lacks practicability, which is underlined by the fact that, to date, ALI has not been implemented in routine diagnostics.

#### A novel biochemical clustering approach

Given the rising number of NCD, there is an urgent necessity to develop an approach that is practicable, cost-efficient, and at best, based on biomarkers that are assessed in clinical routine allowing to identify high-risk individuals to target with specific preventive steps. The current study aimed to develop and validate an easily accessible measure that can realistically be implemented in routine diagnostics. Towards this aim and building on ALI, five biomarkers were chosen as they cover broad physiological functionality; CRP, fibrinogen, and IL-6 are pro-inflammatory markers (i.e., positively associated with inflammation), cortisol as the end product of the hypothalamus–pituitary–adrenal axis is an immune-modulatory mediator playing a crucial role in stress response, and creatinine is important for cellular energy metabolism [15–19]. Contrary to ALI, employing a clustering approach based on these biomarkers allows to account for linear and non-linear interactions among them and to link the resulting clusters to a range of mental and somatic diseases. To examine the association between biochemical clusters and diseases, we focused on depression, heart disease, hypertension, stroke, PUD, and cancer as these represent globally the highest prevalence, the fastest increase in numbers, and the utmost comorbidities [4]. We first clustered biochemical markers and related them to odds ratios (ORs) for diseases in a US population sample and then repeated this process in a Japanese cohort. To ensure representativity, both samples were recruited via random-digit-dialing qualifying them for studies with results generalizable to the population. Towards our aim to ensure that the selected biomarkers and their clustering demonstrate robust applicability across different cultures and ethnicities [20], we chose one US American and one Japanese sample to generate and validate the biochemical clusters.

### Methods

#### Collection of biosamples and the assessment of biochemical markers

##### MIDUS

Blood samples were collected after overnight fasting for the assessment of CRP, IL-6, and fibrinogen, according to the manufacturer guidelines (Dade Behring Inc., Deerfield, IL for CRP and fibrinogen; R&D Systems, Minneapolis, Minnesota for IL-6) [20]. Plasma levels of CRP and fibrinogen were assayed using immunonephelometric assay; IL-6 was quantitatively assessed using enzyme-linked immunosorbent assay (ELISA). The laboratory inter-assay coefficient of variance was 5.7% for CRP, 13% for IL-6, and 2.6% for fibrinogen, all below the 20% acceptable range [21].

To obtain a cumulative cortisol and creatinine measure, 12-h overnight urine samples were collected between 7 PM and 7 AM. Enzymatic colorimetric assays and liquid chromatography-tandem mass spectrometry were performed at the Mayo Medical Laboratory in Rochester, Minnesota. Data were excluded if participants had a renal failure or severe renal decline according to glomerular filtration rate [21].

##### MIDJA

CRP, IL-6, and fibrinogen were assessed analogically to MIDUS, while cortisol was assessed in saliva (3 subsequent days, three times each day) and creatinine was assessed in blood. The 9 saliva measurements were averaged and used as a representative marker for cortisol concentrations [22]. We used blood levels of creatinine.

#### Diseases

Depression, heart disease, hypertension, stroke/transient ischemic attack (TIA), PUD, and cancer were assessed via self-report. Participants were asked if they were ever diagnosed with any of these diseases before/at the time of study participation.

#### Statistical analyses

First, the potential collinearity of the biomarker levels was assessed by calculating Pearson correlations among CRP, fibrinogen, IL-6, creatinine, and cortisol. After randomizing the order of participants [23], we performed a *k*-mean cluster analysis with these markers in the MIDUS sample using IBM SPSS Statistics 27. To ensure the stability of clusters, we repeated the clustering process in subsamples [23]: Specifically, we conducted a median split based on age and performed the clustering for each group separately to assess whether the clusters are age-dependent. For the same purpose, we repeated the clustering procedure after excluding participants with a BMI outside the health range (below 18 or above 35). The next step was to repeat biochemical clustering that was performed for the whole MIDUS sample, in the MIDJA cohort. Finally, *z*-tests were used to compare ORs for diseases among clusters.

### Results

#### Preliminary analyses

In both MIDUS and MIDJA samples, biomarkers were positively correlated (see SI Tables 4 and 5).

In MIDUS, 24.1% of the participants (currently or previously) had depression, 11.5% heart disease, 37.1% hypertension, 4.3% stroke/TIA, 5.3% PUD, and 13.6% cancer. In MIDJA, 4.5% of the participants had depression, 5.6% heart disease, 19.3% hypertension, 1.1% stroke/TIA, 8.3% PUD, and 5.1% cancer.

#### K-mean clustering

**Fig. 2:**
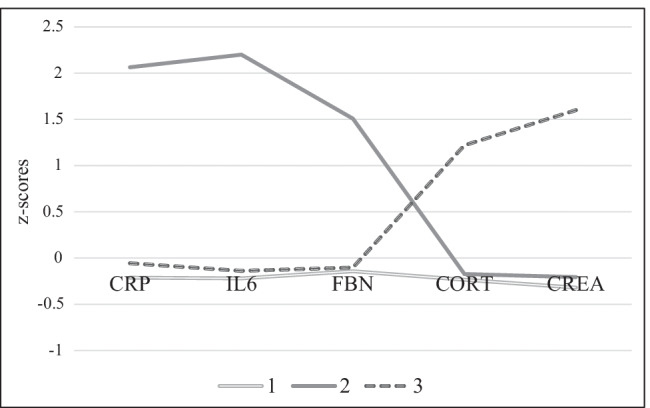
MIDUS: biochemical markers (*z*-scores) and resulting clusters 1–3. *Note*: CRP = C-reactive protein (µg/mL), IL-6 = interleukin-6 (pg/mL), and FBN = fibrinogen (mg/dL) were measured in blood, and cortisol (µg/dL) and creatinine (mg/dL) were measured in urine. *N*_cluster1_ = 937, *N*_cluster2_ = 102, *N*_cluster3_ = 195

We used *z*-standardized biomarkers for *k*-mean clustering and evaluated the clustering results from *k* = 2 to 6 clusters for MIDUS. When *k* = 2, the patterns of clusters were not distinct enough; when *k* = 4 or above, some clusters were very small in size. Through a combination of the parsimonious principle and engineering meaningful difference among clusters, *k* = 3 were selected for the subsequent analyses. Figure 2 illustrates the distributions of the three identified clusters with respect to the biochemical markers. We replicated all three clusters in the younger MIDUS cohort as well as clusters 1 and 2 in the older MIDUS cohort (SI Figs. 7 and 8). We further replicated all three clusters in the BMI-restricted MIDUS cohort (SI Fig. 9).

**Fig. 3:**
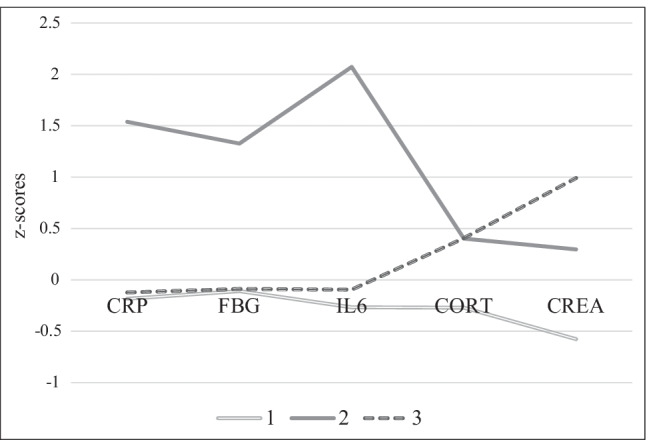
MIDJA: biochemical markers (*z*-scores) and resulting clusters 1–3. *Note*: CRP = C-reactive protein (µg/mL), IL-6 = interleukin-6 (pg/mL) and FBN = fibrinogen (mg/dL), and creatinine (mg/dL) were measured in blood, and cortisol (µg/dL) was measured in saliva. *N*_cluster1_ = 233, *N*_cluster2_ = 30, *N*_cluster3_ = 115

Then, the 3-cluster solution from MIDUS was validated in the MIDJA sample; the results are shown in Fig. 3.

As depicted in Figs. 2 and 3, cluster 1 is characterized by average levels in all biochemical measures. Cluster 2 is characterized by high and above-average levels for CRP, IL-6, and fibrinogen. Cluster 3 is characterized by high and above-average levels for cortisol and creatinine but average concentrations of CRP, fibrinogen, and IL-6.

#### Associations between biochemical clusters and disease states

##### MIDUS

**Fig. 4:**
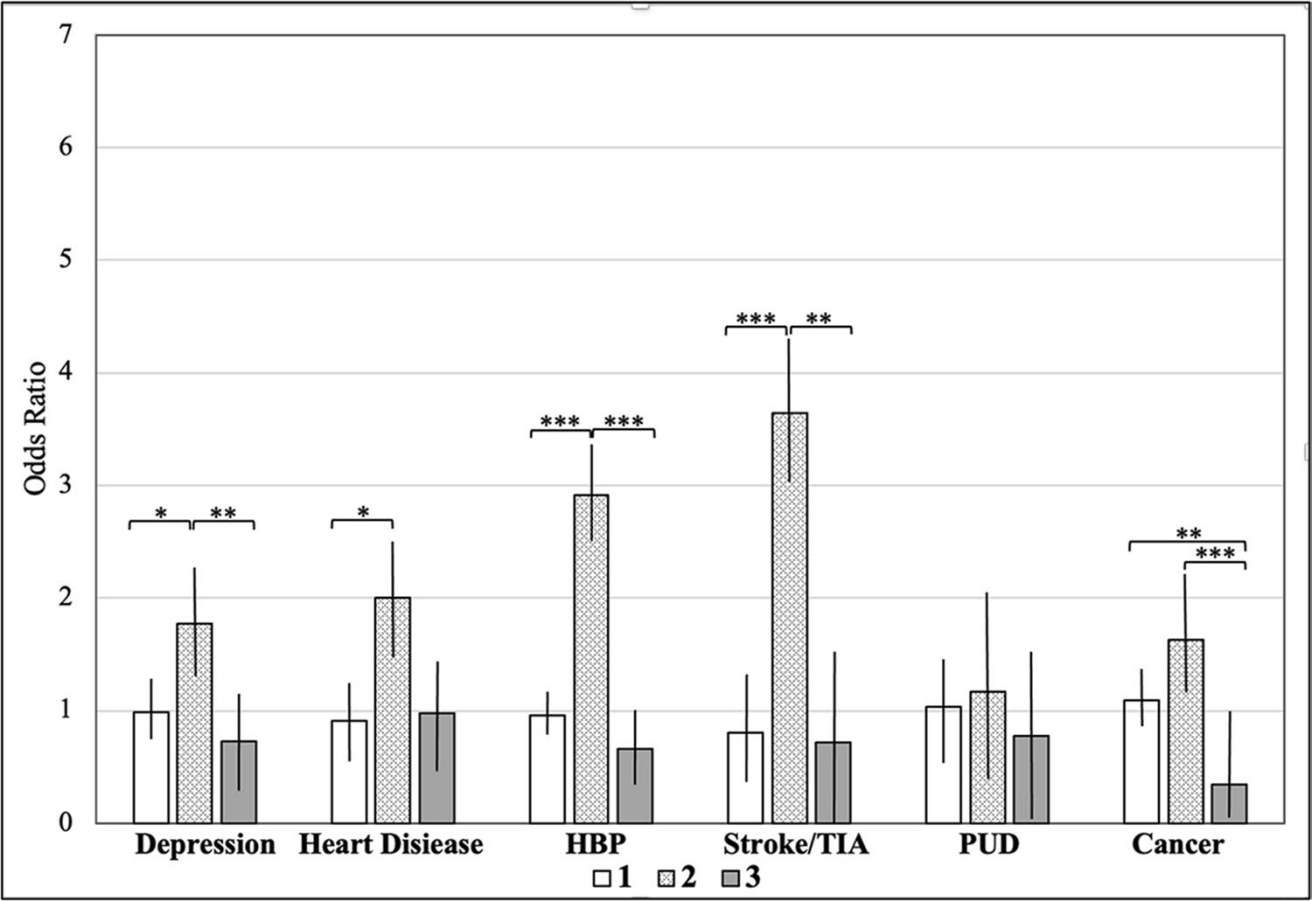
MIDUS: odds ratios for diseases by cluster. *Note*: MIDUS = Midlife in the US sample, HPB = high blood pressure, TIA = transient ischemic attack, PUD = peptic ulcer disease. Error bars display 95% confidence intervals. Comparisons of odds ratios were conducted with log odds ratios using *z*-tests. * *p* < .05, ** *p* < .01, *** p < .001, *p*-values are controlled for multiple testing according to Bonferroni. All two-tailed

Cluster 2 had the highest ORs for all considered diseases compared to the clusters 1 and 3 (Fig. 4, SI 10).

##### MIDJA

**Fig. 5:**
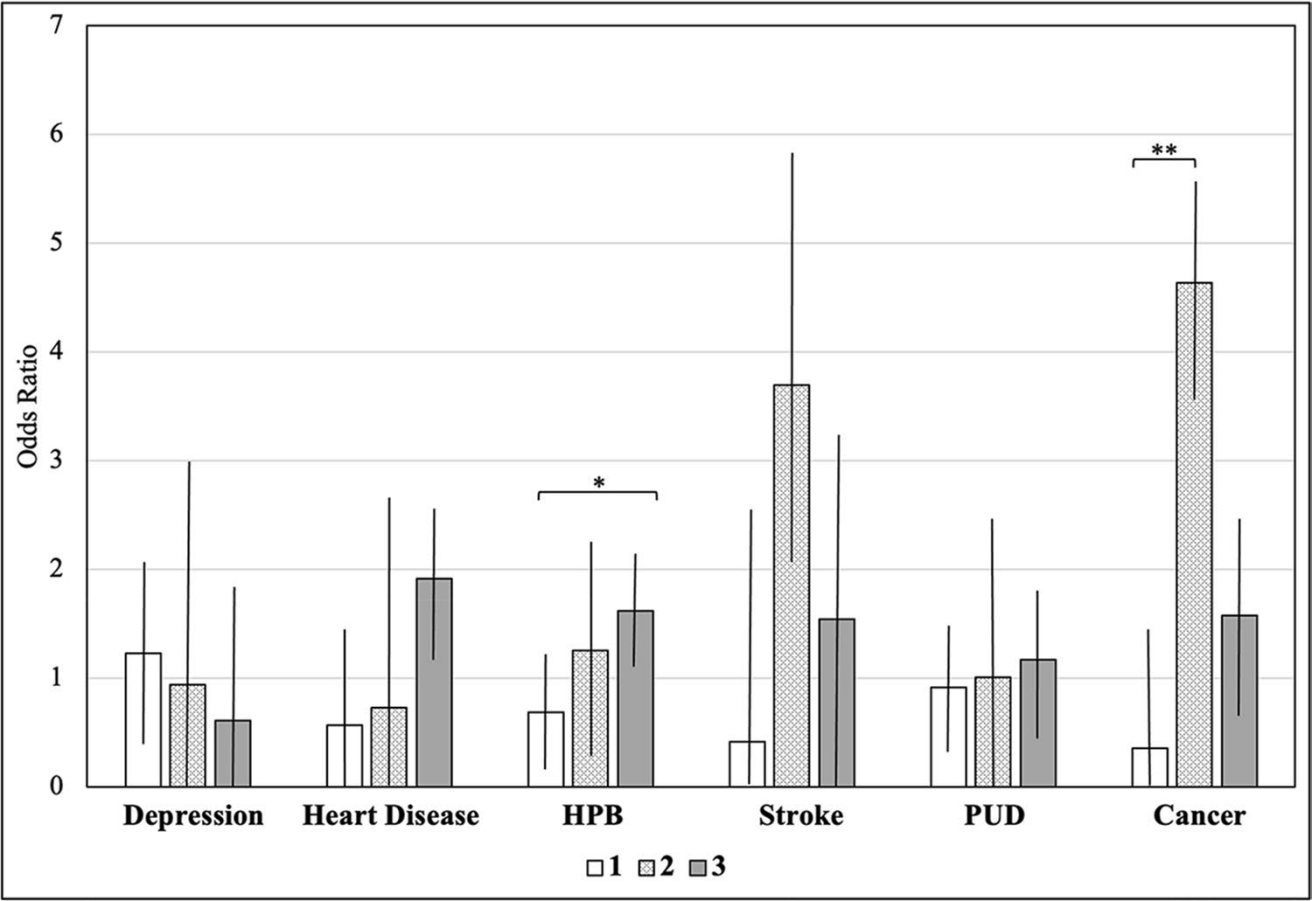
MIDJA: odds ratios for diseases by cluster. *Note*: MIDJA = Midlife in Japan sample, HPB = high blood pressure, TIA = transient ischemic attack, PUD = peptic ulcer disease. Error bars display 95% confidence intervals. Comparisons of odds ratios were conducted with log odds ratios using *z*-tests. * *p* < .05, ** *p* < .01, *** p < .001, *p*-values are controlled for multiple testing according to Bonferroni. All two-tailed

Cluster 3 had the highest ORs for heart disease, hypertension, and PUD, cluster 2 had the highest ORs for stroke and cancer, and cluster 1 had the highest ORs for depression (Fig. 5, SI 10.1).

To compare this cluster-based approach to a well-established clinical biomarker that is associated with a broad range of NCD, the number of diagnoses among individuals in cluster 2 was compared to the number of diagnoses among individuals with CRP concentrations above the clinical cutoff (> 3 mg/L) [24]. The disease burden in cluster 2 was higher with 1.6 diagnoses (*SD* = 1.16; 0.9 diagnoses for individuals not assigned to cluster 2) compared to individuals above the CRP cutoff with 1.2 diagnoses (*SD* = 1.07; 0.9 diagnoses for individuals below the cutoff).

### Discussion

#### Three biochemical clusters in the general population

Findings reveal three distinct and interculturally stable biochemical clusters observable in the general population. Cluster 1 is characterized by average levels of all biomarkers, cluster 2 by high inflammation-related mediators coupled with low cortisol and creatinine, and cluster 3 by high levels of cortisol and creatinine. The stability of clusters is supported by their replication in the MIDJA sample as well as in the BMI-restricted, in the younger (below age median) and in the older MIDUS cohort (above age median; here only clusters 1 and 2 were replicated). However, we did not replicate cluster 3 in the older MIDUS cohort. One explanation could be that, due to an age-related increase in systemic inflammation [25], older individuals were not assigned to cluster 3, which is characterized by low inflammation.

#### The link of biochemical clusters to disease states

Relating clusters to diseases, in MIDUS, cluster 2 showed the highest ORs for depression, heart disease, hypertension, stroke, and cancer (Fig. 4). These findings are supported by previous evidence suggesting that CRP, IL-6, and fibrinogen are associated with depression [26, 27], coronary heart disease [28–31], blood pressure [32], stroke [33–35], and cancer [36, 37]. However, contrary to these previous studies, the clustering approach used in this study allowed to account for well-known collinearities between biomarkers and thus promotes a more holistic perspective. Specifically, findings build on previous studies suggesting a link between inflammation and diseases [25] by demonstrating that it might not be one specific biomarker but a specific biochemical pattern (i.e., high CRP, IL-6, fibrinogen coupled with low cortisol and creatinine) that is associated with diseases. This idea is supported by the observation that individuals in cluster 2, descriptively, indicate a higher disease burden than individuals above the clinically well-established CRP cutoff.

Interestingly, we found no differences in the ORs for PUD between clusters despite the role of inflammation in its pathology [38]. Future research may aim to further examine the role of inflammatory signaling in the pathology of PUD.

While the cluster with high levels of CRP, IL-6, and fibrinogen can be considered a high-risk cluster, cluster 3 with high levels of cortisol and creatinine but low inflammation may be considered a protective cluster in MIDUS. We found that ORs for most diseases were lower in cluster 3 not only as compared to the high-risk cluster but also as compared to cluster 1 with average levels of all biomarkers. Concerning cancer, this difference became significant, potentially suggesting a protective character of this cluster. This would be in contrast to studies suggesting a link between hypercortisolism and disease outcomes [39, 40]. However, the combination of low inflammation and high cortisol and creatinine as in cluster 3 might indicate the integrity of the glucocorticoid negative feedback system, protecting from negative health outcomes [41]. Longitudinal studies may examine the consequences of this specific biochemical pattern. Towards this aim, we will examine MIDUS follow-up data (10 years after biomarker assessments) with respect to mortality outcomes.

In MIDJA, cluster 2 only seems to be a high-risk cluster for stroke and cancer while for other considered diseases, cluster 1 or cluster 3 indicates the highest burden. One aspect to consider here is that the MIDJA sample (*N* = 378) and especially cluster 2 were very small in size (*N* = 30). It is, therefore, possible that the present findings lack reliability. However, different biochemical patterns may be associated with different outcomes in the Japanese compared to the US American population because moderating mechanisms such as BMI, nutrition, and medication differ between populations [41]. This idea is supported by the finding that although in both MIDUS and MIDJA, approximately 8% of participants were assigned to cluster 2, the disease burden in MIDJA was much lower compared to MIDUS. This highlights the importance of individual aspects in disease susceptibility mentioned above and the role of interactions among different cultural, lifestyle, and biochemical factors; while an assignment of a US American individual to cluster 2 might be associated with a high disease burden, this might not be the case for a Japanese individual with the similar biochemical profile. Future studies should aim to examine the found biochemical clusters in other cultural contexts promoting a better understanding of their associative and predictive character in multiple populations. From a preventive perspective, this may also help to further precise targeted prevention, that is, to better understand which biochemical profile is associated with what disease susceptibility under what conditions.

#### Limitations

Our work has several strengths such as the validation of the clusters in an independent, Japanese sample and the representative character of cohorts. Yet, the findings face limitations. First, the present study is cross-sectional not allowing causal inferences. Second, the MIDJA sample size was relatively small. It is, therefore, possible that the ORs lack reliability. Third, methodological inconsistencies (urine cortisol and creatinine levels in MIDUS, average saliva levels of cortisol and blood levels of creatinine in MIDJA) between the cohorts may have impacted the clustering process. Fourth, diseases were assessed via self-report, which bears the risk of a report bias.

#### Conclusion

While the interactions among biomarkers make the distinction of their outcomes challenging, the design of the current study helps to gain a better understanding regarding the biochemical patterns that are present in the general population and how these patterns contribute to different physiological states on a systemic scale. We identified and replicated three distinct biochemical signatures in two mid-life populations including one cluster with collinearly occurring elevated levels of CRP, fibrinogen, and IL-6 as well as low concentrations of cortisol and creatinine that indicated the highest prevalence of stroke and cancer.

Future longitudinal studies should aim to test the predictive character of the clusters found in this study, because, if clusters are indeed predictive in terms of risk evaluation, then they would represent a valuable clinical tool for both diagnostics and prevention of diseases. Specifically, if high-risk individuals can be identified by the clustering approach presented here, then these individuals could be provided with personalized treatment options including psychotherapy, anti-inflammatory drugs, and treatment supplements, e.g., nutrition and exercise plans.

## Study 2

### Introduction

#### The role of childhood maltreatment in disease susceptibility

Childhood maltreatment (CM) is an umbrella term that includes any act of emotional, physical, and sexual abuse as well as emotional and physical neglect experienced until the age of 18 [42]. CM can have a myriad of negative effects on survivors’ mental and somatic health. The association between CM and inflammation is well established and may underlie the increased prevalence of somatic and mental disorders in CM-exposed individuals [16, 43–45]. Thus, CM, which is still an underestimated phenomenon in somatic/clinical settings, might be a disruptive factor in the context of both personalized medicine and targeted prevention, as it may amplify and interact with other disease susceptibility factors, resulting in a massive increase and expansion of an individual’s disease risk and development. Therefore, in study 2, the association of CM with disease prevalence as well as with the assignment to the biochemical clusters was investigated.

We used the MIDUS sample for these analyses, as CM was not assessed in MIDJA. Based on previous literature, we expected to find higher exposure of CM in clusters with high inflammation as compared to clusters with low inflammation [16, 43–45].

### Methods

#### Assessment of childhood maltreatment

CM was assessed using the Childhood Trauma Questionnaire (CTQ; Bernstein and Fink [46]). As a retrospective self-report measure with 28 items, the CTQ assesses five types of CM: emotional, physical, and sexual abuse, emotional, and physical neglect as well as the tendency to minimize CM [46].

#### Statistical analyses

Cutoff values for moderate CM exposure were used to create dichotomous variables for each CTQ subscale (emotional abuse ≥ 13; physical abuse ≥ 10; sexual abuse ≥ 8; emotional neglect ≥ 15; and physical neglect ≥ 10) [46]. A composite variable was then computed indicating exposure to at least one category of moderate to severe abuse or neglect (CM +) vs. no or low exposure (CM −) [46]. Using the moderate cutoff variable, prevalences of CM were calculated for the whole sample. Next, we compared general disease burden as well as the prevalence of specific diseases in individuals without and with CM experiences using *χ*^2*−*^tests and *t*-tests. Then, a continuous total score of the CTQ was calculated by summing up the scores across all items. This continuous score was used to create a general linear model (GLM) with pairwise comparisons correcting for sex, age, BMI, physical activity, alcohol, and smoking habits as well as for multiple testing (Bonferroni) comparing CM among clusters. To avoid issues resulting from heteroscedastic residual variances, we performed a bootstrapping (10,000 samples). Bootstrapping, which allows finding robust parameter estimates (i.e., independently from the homoscedasticity assumption of residual variances), is considered the gold standard approach since our clusters are stable and since none of the covariates included in the GLM is involved in the clustering process [47].

### Results

One-third (36.1%) of participants reported at least moderate CM on at least one CTQ subscale. Individuals exposed to CM had a higher overall disease burden with 1.12 (*SD* = 1.03) diagnoses on average compared to 0.85 (*SD* = 0.93) diagnoses in individuals without CM history (*t*(1192) =  − 4.549, *p* < 0.001). This difference was mainly driven by the higher prevalence of depression in CM-exposed individuals (36.2%) compared to individuals without CM (16.9%, *χ*^2^(1) = 61.72, *p* < 0.001).

CM exposure differed between biochemical clusters, with 45.1% of individuals in cluster 2 reporting at least moderate CM on at least one of the CTQ subscales (28.4% without CM), compared to 35.9% in cluster 1 (37.1% without CM) and 30.8% in cluster 3 (43.6% individuals without CM). GLMs using the continuous CM score indicated (SI Table 13) the highest CM exposure in cluster 2, followed by clusters 1 and 3 (all *p*s < 0.001).

### Discussion

#### Childhood maltreatment and disease burden: a mediating role for biochemical profiles?

The CM prevalences found here are in line with meta-analytic findings [48] as well as the result that CM-exposed individuals have a higher disease burden compared to non-exposed individuals is supported by previous evidence [49–51]. Given the association of CM to inflammatory processes [16, 43–45], one mechanism possibly linking CM to diseases might be the biochemical clusters from study 1. As we found that cluster 2 had the highest CM exposure and also the highest disease prevalences, specific biochemical profiles may underlie the association between CM and disease burden. If that is the case, clusters may represent a future leverage point for targeted prevention, enabling CM-exposed individuals to overcome the abusive experience and their stress burden-related health consequences through e.g. psychotherapy and support groups before it comes to the onset and manifestation in the form of severe disease. However, this idea faces the limitation that we could not statistically test this mediation of the biochemical clusters in the link between CM and disease prevalences as both the possible mediator (clusters) and the dependent variables (disease yes/no) were categorial. To get a deeper insight into this issue, our aim with the MIDUS follow-up data (10 years after biomarker assessments) is to examine whether CM-exposed individuals in cluster 2 indeed show more detrimental outcomes than CM-exposed individuals in the other two clusters.

#### Limitations

The present findings should be considered in light of the limitation that we used retrospective self-reported measures of CM. Therefore, report and memory biases are possible. Although the value of self-reported measures of CM when investigating its correlates and outcomes has been emphasized [52], future studies should also aim to relate CM assessed via official reports to the found biochemical clusters and to diseases. CM was not available in the Japanese cohort; therefore, the associative nature of CM with the identified clusters in the US sample needs future replication in independent cohorts. As this study was cross-sectional, causal inferences cannot be drawn without subsequent research.

#### Conclusions

Findings complement existing literature indicating detrimental longer-term implications of CM on survivors’ health. Results highlight the importance of identifying CM as early as possible before it manifests itself biologically and possibly increases disease vulnerability. We thus encourage professionals in preventive and medical care contexts to be attentive to reports of CM and to consider these in individual treatments; validated screening instruments are available in multiple languages (e.g., CTQ) [46].

## Conclusions and expert recommendations in the framework of 3P medicine

### The contribution of the current findings

Our findings suggest three distinct biochemical signatures that are replicable and interculturally stable. One of them is a high-risk cluster indicated by its high disease burden. Due to the cross-sectional character of this study, it might also be that the biochemical clusters are consequences of diseases; however, study 2 demonstrating a strong link between the high-risk cluster and CM provides first hints that the clusters could be indeed pre-disease markers affecting the vulnerability to diseases. Future studies should aim to test the predictive character of clusters to evaluate their applicability as pre-disease markers. Furthermore, integrating CM screenings in standard medical practice may be a promising way for identifying individuals at risk and for developing tailored prevention and intervention techniques.

### Implications and recommendations for personalized medicine, targeted prevention, and predictive diagnostics

The assessment of CRP, IL-6, fibrinogen, cortisol, and creatinine should be mandatory in all 3PM (i.e., personalized medicine, targeted prevention, and predictive diagnostics) disciplines to get a global insight into an individual’s current health condition. High inflammatory signaling coupled with low compensation, that is, with low cortisol and creatinine, is a detrimental biochemical profile associated with a high disease burden and should be taken as a reason for further examination (especially with respect to artery condition/stroke and cancer) and for personalized treatments involving anti-inflammatory drugs, nutrient substitutions, and treatment supplements, e.g., nutrition and exercise plans. Furthermore, individuals with this biochemical profile should be examined with a special focus on early-life stress and especially CM. In cases where CM is prevalent, its role in the patient’s individual condition pattern should be examined thoroughly and psychotherapy or other stress reducing interventions should be offered/employed.

### Future research directions to foster the understanding of biochemical profiles in personalized medicine, targeted prevention, and predictive diagnostics

Future research should examine the predictive character of the found biochemical clusters with respect to long-term well-being, mental and physical health, and mortality. Ideally, these studies should examine different cultures promoting a better understanding of the generalizability and limitedness of the predictive power of the identified biochemical clusters. Furthermore, this future research may suggest additional factors to be taken into account together with the biochemical clusters, helping to advance and precise disease prediction and, hence, to improve both targeted prevention and personalized interventions.

## Supplementary Information

Below is the link to the electronic supplementary material.

Supplementary file1 (DOCX 61 KB)

## Data Availability

All analyzed data are available publicly: http://www.midus.wisc.edu/data/index.php.
