# Supplementary material for: How biomarker patterns can be utilized to identify individuals with a high disease burden: a bioinformatics approach towards predictive, preventive, and personalized (3P) medicine
Source: EPMA J. 2021 Sep 29;12(4):507–16. doi: 10.1007/s13167-021-00255-0 (PMC8648886; doi:10.1007/s13167-021-00255-0)
Supplement: Supplementary file 1 — Supplementary file1 (DOCX 61 KB) [file 13167_2021_255_MOESM1_ESM.docx]

**How Biomarker Patterns can be Utilized to Identify Individuals with a High Disease Burden: A Bioinformatics Approach Towards Predictive, Preventive and Personalized (3P) Medicine**

**Supplemental Material**

**Study 1**

**S1. MIDUS: Additional Information on Setting and Procedure.**

For the first study sample, i.e., MIDUS, a total of  *N* = 7,108 individuals between 25 and 74 years of age were recruited from January 1995 to September 1996 from a national random-digit-dial sample of non-institutionalized adults living in the 48 contiguous states [1]. Participants from MIDUS were recruited again for a follow-up study (2004-2006) focusing on biomarkers, namely MIDUS2, yielding a 70% response rate (*N* = 4,963). Additionally, a supplement sample of African Americans (*N* = 592) was recruited from Milwaukee, Wisconsin. A total of 1,255 individuals participated in the biomarker study, and of those complete biomarker data (regarding CRP, IL-6, fibrinogen, cortisol, and creatinine) was available from 1,234 individuals. Biomarker data collection was carried out at three General Clinical Research Centers (at UCLA, University of Wisconsin, and Georgetown University). The institutional review boards at each university approved all data collection [1].

**S2. Additional Information and Justification of K-mean Clustering Approach.**

K-mean clustering is a commonly used approach to classify multidimensional data into groups with specific patterns, and has previously been used in analysis of phenotypes based on biochemical markers [2]. K-mean cluster analysis panels the data points into a pre-defined number (k) of the clusters . An observation is assigned to the nearest cluster measured by Euclidean distance [2].

K-mean cluster analysis was used to identify distinct biochemical patterns. The reason to prefer a k-mean generated cluster variable before the original biochemical indicator variables include that first, k-mean generated clusters provide discrete memberships of biochemical patterns, which is a primary goal of this study and second, that k-mean generated cluster variables can alleviate multicollinearity issues faced by directly using the original biomarkers in a regression model and can account for linear and non-linear interactions among the original variables.

| **Table S3.** Demographics for Whole Sample and by Cluster. | | | |
| --- | --- | --- | --- |
|  |  | MIDUS | MIDJA |
| Total sample | Sex | 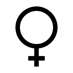=56.8%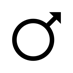=43.2% | 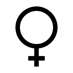=56.1% 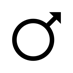=43.9% |
|  | Age | *M*=52.52 *SD*=11.71 | *M*=55.34 *SD*=14.02 |
|  | BMI | *M*=29.77 *SD*=6.626 | *M*=22.6 *SD*=2.96 |
|  | Activity | 76.5% | N/A |
|  | Alcohol | *M*=3.76 *SD*=1.4 | N/A |
|  | Smoking | 47.6% | N/A |
| Cluster 1 | Sex | 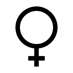=76.2%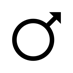=23.8% | 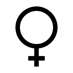=75.6%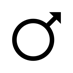=24.4% |
|  | Age | *M*=55.25 *SD*=11.61 | *M*=53.31 *SD*=13.51 |
|  | BMI | *M*=28.88 *SD*=5.81 | *M*=22.07 *SD*=3 |
|  | Activity | 79.5% | N/A |
|  | Alcohol | *M*=3.71 *SD*=1.42 | N/A |
|  | Smoking | 47% | N/A |
| Cluster 2 | Sex | 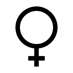=59.6%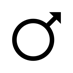=40.4% | 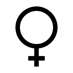=48.1%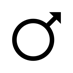=51.9% |
|  | Age | *M*=55.40 *SD*=12.09 | *M*=65 *SD*=11.87 |
|  | BMI | *M*=34.49 *SD*=9.26 | *M*=23.77 *SD*=2.64 |
|  | Activity | 57.8% | N/A |
|  | Alcohol | *M*=4.15 *SD*=1.32 | N/A |
|  | Smoking | 55.9% | N/A |
| Cluster 3 | Sex | 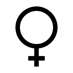=31.3%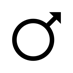=68.7% | 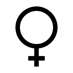=23%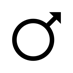=77% |
|  | Age | *M*=50.63 *SD*=11.24 | *M*=56.87 *SD*=14.44 |
|  | BMI | *M*=30.9 *SD*=6.99 | *M*=23.32 *SD*=2.75 |
|  | Activity | 72.8% | N/A |
|  | Alcohol | *M*=3.78 *SD*=1.34 | N/A |
|  | Smoking | 45.6% | N/A |
| *Note:* MIDUS=Midlife in the United States study, MIDJA= Midlife in Japan Study, BMI=Body Mass Index, Activity: Do you engage in regular exercise, or activity, of any type for 20 minutes or more at least 3 times/week? (1=yes, 2=no), Alcohol: Past month, how often did you drink any alcoholic beverages, on average? (1=everyday, 2=5 or 6 days/week, 2=3 or 4 days/week, 4=1 or 2 days/week, 5=less than one day/week, 6=Never drinks), Smoking: Ever smoked cigarettes regularly? (1=yes, 2=no). | | | |

| **Table S4.** MIDUS: Descriptive Statistics and Correlations Among Biochemical Markers. | | | | | |
| --- | --- | --- | --- | --- | --- |
|  | CRP | IL-6 | Fibrinogen | Cortisol | Creatinine |
| *Mean*  *SD* | 3.03  4.78 | 3.04  3.05 | 348.92  87.85 | 1.09  1.13 | 81.24  53.7 |
| CRP | - |  |  |  |  |
| IL-6 | .39^***^ | - |  |  |  |
| Fibrinogen | .49^***^ | .36^**^ | - |  |  |
| Cortisol | .08^**^ | -.03 | -.02 | - |  |
| Creatinine | .05 | .01 | -.06 | .38^***^ | - |
| *Note:* CRP=C-reactive protein (ug/mL), IL-6=Interleukin-6 (pg/mL) and fibrinogen (mg/dL) were measured in blood, cortisol (ug/dL) and creatinine (mg/dL) were measured in urine.  ** *p*<.01, *** *p*<.001, *p*-values are controlled for multiple testing according to Bonferroni.  All two-tailed. | | | | | |

| **Table S5.** MIDJA: Descriptive Statistics and Correlations Among Biochemical Markers. | | | | | |
| --- | --- | --- | --- | --- | --- |
|  | CRP | IL-6 | Fibrinogen | Cortisol | Creatinine |
| *Mean*  *SD* | .67  1.28 | 1.55  1.68 | 319.06  64.2 | 7.43  2.78 | .74  .17 |
| CRP | - |  |  |  |  |
| IL-6 | .51^***^ | - |  |  |  |
| Fibrinogen | .38^***^ | .26^**^ | - |  |  |
| Cortisol | .12 | .08 | .05 | - |  |
| Creatinine | .15^*^ | .2^***^ | .09 | .07 | - |
| *Note:* CRP=C-reactive protein (ug/mL), IL-6=Interleukin-6 (pg/mL), fibrinogen (mg/dL) and creatinine (mg/dL) were measured in serum, cortisol was measured in saliva.  ** *p*<.01, *** *p*<.001, *p*-values are controlled for multiple testing according to Bonferroni.  All two-tailed. | | | | | |

**S6. Additional Stability Analyses of MIDUS clusters (see S7-9).**

To ensure the stability of clusters, we conducted additional analyses with MIDUS as this was the larger sample. More specifically, we did a median-split based on age and performed the clustering for each group separately to see whether the clusters are age-dependent. We further performed the clustering for the whole MIDUS sample again after excluding adult participants with a BMI outside the health range (i.e., below 18 or above 35) to see whether the clustering was influenced by extreme BMI.

**Figure S7.** MIDUS Young Sample: Biochemical Markers (z-scores) by Cluster.

*Note:* CRP=C-reactive protein (ug/mL), IL-6=interleukin-6 (pg/mL) and FBN=fibrinogen (mg/dL) were measured in blood, cortisol (ug/dL) and creatinine (mg/dL) were measured in urine. Only participants below the age median (i.e., 54 years) were included. *N_total_* = 624, *N_cluster1_* = 435, *N_cluster2_* = 59, *N_cluster3_* = 130.

**Figure S8.** MIDUS Old Sample: Biochemical Markers (z-scores) by Cluster.

*Note:* CRP=C-reactive protein (ug/mL), IL-6=interleukin-6 (pg/mL) and FBN=fibrinogen (mg/dL) were measured in blood, cortisol (ug/dL) and creatinine (mg/dL) were measured in urine. Only participants above the age median (i.e., 54 years) were included. *N_total_* = 601,

*N_cluster1_* = 370, *N_cluster2_* = 28, *N_cluster3_* = 203. Nine individuals were excluded due to CRP and/or cortisol levels more than 5 standard deviations above the mean.

**Figure S9.** MIDUS BMI-Adjusted Sample: Biochemical Markers (z-scores) by Cluster.

*Note:* CRP=C-reactive protein (ug/mL), IL-6=interleukin-6 (pg/mL) and FBN=fibrinogen (mg/dL) were measured in blood, cortisol (ug/dL) and creatinine (mg/dL) were measured in urine. Participants with a Body Mass Index (BMI) below 18 and above 35 were excluded.

*N_total_* = 1,004, *N_cluster1_* = 737, *N_cluster2_* = 63 *N_cluster3_* = 204.

**S10. MIDUS: Relations of Clusters to Diseases.**

According to our *z*-tests, cluster 2 showed the highest odds ratios for depression (*z*=-2.381, *p*=.026 for balanced vs. high risk; *z*=3, *p*=.004 for mixed vs. high-risk), for high blood pressure (HBP; *z*=-2.68, *p*<001 for balanced vs. high-risk; *z*=2.02, *p*<.001 for blue vs. high-risk), and stroke (*z*=-3.84, *p*<.001 for balanced vs. high-risk; *z*=2.98, *p*=.004 for mixed vs. high-risk). With respect to heart disease, cluster 2 had significantly higher odds ratios as compared to cluster 1 (*z*=-2.68, *p*=.011), and regarding cancer cluster 2 had significantly higher odds ratios as compared to cluster 3 (*z*=3.69, *p*<.001) while cluster 1 had significantly higher odds ratios as compared to cluster 3 (*z*=3.24, *p*=.002). With respect to PUD, we found no significant differences in the odds ratios across clusters.

**S10.1 MIDJA: Relations of Clusters to Diseases.**

Our *z*-tests demonstrated that cluster 2 had significantly higher odds ratios for cancer as compared to cluster 1 (*z*=-3.25, *p*=.002), and cluster 1 had significantly higher odds ratios for HBP (*z*=-2.52, *p*=.018) as compared to the mixed cluster.

**Study 2**

**S11. Childhood Trauma Questionnaire**

In general, participants are asked to rate items of the CTQ on a 5-point Likert-type scale with response options ranging from 1 (*never true*) to 5 (*very often true*). Bernstein and Fink (1998) suggest building a sum score of all items of the subscales with higher scores representing higher exposure to CM. Furthermore, it is common to build cutoff scores based on the CTQ subscales, e.g., a mild cutoff differentiating individuals with no or minimal CM experiences from individuals with significant CM experiences on at least one CTQ subscale (i.e., sum score >8 for emotional abuse, >7 for physical abuse, >5 for sexual abuse, >9 for emotional neglect, or >7 for physical neglect) [3]. Cronbach’s alpha in the current study was .95.

| **Table S12.** MIDUS: Descriptive Statistics and Correlations Among Childhood Maltreatment Types and Biochemical Markers. | | | | | | | | | | | | |
| --- | --- | --- | --- | --- | --- | --- | --- | --- | --- | --- | --- | --- |
|  | | CM | EA | PA | SA | EN | PN | CRP | IL-6 | FIB | CORT | CREA |
| *Mean* |  | 38.3 | 8.05 | 6.99 | 6.61 | 9.77 | 6.91 | 3.02 | 3.04 | 348.92 | 1.09 | 81.24 |
| *SD* |  | 14.53 | 4.22 | 3.06 | 3.98 | 4.57 | 2.77 | 4.78 | 3.04 | 87.85 | 1.13 | 53.7 |
| CM |  | - | .870^**^ | .788^**^ | .657^**^ | .827^**^ | .741^**^ | .064^*^ | .077^**^ | .092^**^ | -.09^**^ | .005 |
| EA |  | .87^**^ | - | .688^**^ | .441^**^ | .678^**^ | .531^**^ | .041 | .036 | .048 | -.0^**^ | -.028 |
| PA |  | .79^**^ | .69^**^ | - | .430^**^ | .518^**^ | .511^**^ | .034 | .040 | .093^**^ | -.082 | .045 |
| SA |  | .66^**^ | .44^**^ | .43^**^ | - | .319^**^ | .335^**^ | .085^**^ | .080^**^ | .096^**^ | -.061^*^ | -.033 |
| EN |  | .82^**^ | .68^**^ | .52^**^ | .32^**^ | - | .627^**^ | .036 | .048 | .046 | -.054 | .021 |
| PN |  | .74^**^ | .53^**^ | .51^**^ | .34^**^ | .63^**^ | - | .049 | .112^**^ | .101^**^ | -.051 | .030 |
| CRP |  | .06 | .04 | .03 | .09^*^ | .04 | .05 | - | .391^**^ | .492^**^ | .084^**^ | .052 |
| IL-6 |  | .08^*^ | .04 | .04 | .08^*^ | .05 | .11^**^ | .39^**^ | - | .360^**^ | -.028 | .010 |
| FIB |  | .09^*^ | .05 | .09^*^ | .01^*^ | .05 | .10^**^ | .49^**^ | .36^**^ | - | -.020 | -.064^*^ |
| CORT |  | -.09^*^ | -.1^*^ | -.08^*^ | -.06 | -.05 | -.05 | .08^*^ | -.03 | -.02 | - | .384^**^ |
| CREA |  | .01 | -.03 | .05 | -.03 | .02 | .03 | .05 | .01 | -.06 | .38^**^ | - |
| *Note:* CM=childhood maltreatment; refers to the overall score, EA=emotional abuse, PA=physical abuse, SA=sexual abuse, EN=emotional neglect, PN=physical neglect, CRP=C-reactive protein (ug/mL), IL-6=Interleukin-6 (pg/mL), and FIB=fibrinogen (mg/dL) were measured in serum, CORT=cortisol (ug/dL) and CREA=creatinine (mg/dL) were measured in urine. | | | | | | | | | | | | |
| ** p*<.05 ** *p*<.01, *p*-values are controlled for multiple testing according to Bonferroni. | | | | | | | | | | | | |

| **Table S13.** General Linear Model Predicting Childhood Maltreatment By Cluster. | | | | | |
| --- | --- | --- | --- | --- | --- |
| Dependent Variable: CTQ Sum score | | | | | |
| Source | Type III Sum of Squares | df | Mean Square | F | *p* |
| Corrected Model | 1563901920.14^a^ | 709 | 2205785.5 | 123936.67 | <.001 |
| Intercept | 241538242.56 | 1 | 241538242.56 | 13571331.2 | <.001 |
| Cluster | 3019986.43 | 2 | 1509993.22 | 84842.13 | <.001 |
| Gender | 116191.31 | 1 | 116191.31 | 6528.45 | <.001 |
| Age | 96700306.77 | 49 | 1973475.65 | 110883.86 | <.001 |
| BMI | 1327678509.49 | 650 | 2042582.32 | 114766.76 | <.001 |
| Physical activity | 484217.85 | 1 | 484217.85 | 27206.79 | <.001 |
| Alcohol | 3254689.1 | 5 | 650937.82 | 36574.3 | <.001 |
| Smoking | 28830.93 | 1 | 28830.93 | 1619.93 | <.001 |
| Error | 143451028.84 | 8060096 | 17.8 |  |  |
| Total | 13564939758.34 | 8060806 |  |  |  |
| Corrected Total | 1707352948.99 | 8060805 |  |  |  |
| 1. *R^2^*= .92 (*R^2^_adjusted_*= .92)   Bootstrapping was performed using 10,000 samples.  CTQ = Childhood Trauma Questionnaire. | | | | | |

| **Table S13.1.** Pairwise Comparisons Of Childhood Maltreatment  Among Clusters. | | | | | | |
| --- | --- | --- | --- | --- | --- | --- |
| Dependent Variable: CTQ Sum score | | | | | | |
| (I) | (J) | Mean Difference (I-J) | Std. Error | Sig. | 95% Confidence Interval | |
|  |  |  |  |  | Lower Bound | Upper Bound |
| 1 | 2 | -6.92 | .01 | <.001 | -6.93 | -6.9 |
|  | 3 | .68 | .01 | <.001 | .67 | .69 |
| 2 | 1 | 6.92 | .01 | <.001 | 6.9 | 6.93 |
|  | 3 | 7.6 | .01 | <.001 | 7.59 | 7.61 |
| 3 | 1 | -.68 | .01 | <.001 | -.69 | -.67 |
|  | 2 | -7.6 | .01 | <.001 | -7.61 | -7.58 |
| Based on observed means. The error term is Mean Square(Error) = 17.7.  *p*-values are corrected for multiple testing (Bonferroni,  i.e., multiplied by three due to three pairwise comparisons).  Bootstrapping was performed using 10,000 samples.  CTQ = Childhood Trauma Questionnaire. | | | | | | |

**References for the Supplemental Material**

1. Ryff CD, Seeman T, Weinstein M. Midlife in the United States (MIDUS 2): Biomarker Project, 2004-2009: Version 9 [Internet]. Inter-University Consortium for Political and Social Research; 2010 [cited 2021 Mar 27]. Available from: https://www.icpsr.umich.edu/icpsrweb/NACDA/studies/29282/versions/V9

2. Franklin J. The elements of statistical learning: data mining, inference and prediction. The Mathematical Intelligencer. 2005;27:83–5.

3. Bernstein D, Fink L. Manual for the childhood trauma questionnaire. The Psychological Corporation. 1998;
